# First, you need a Gestalt: An interaction of bottom-up and top-down streams during the perception of the ambiguously rotating human walker

**DOI:** 10.1038/s41598-017-01376-1

**Published:** 2017-04-25

**Authors:** Alexander Pastukhov

**Affiliations:** Otto-Friedrich-Universität, Department of General Psychology and Methodology, Bamberg, 96047 Germany

## Abstract

Our visual system combines sensory evidence with prior knowledge to produce a representation of an outside world. Here, we explored the limits of the feedforward computation using an ambiguously rotating human biological motion. Specifically, we investigated whether an overall rotation, which was added to all the displays used in the study, would be perceived when the point-light walker was presented upside-down, a condition that typically obliterates perception of a human Gestalt. We report that inversion of the point-light walker or the absence of an identifiable Gestalt abolished the perception of an overall rotation. Perception of rotation was restored if the human walker Gestalt could be identified (an upright walker), if observers were informed about the nature of the motion display, or if observers expected to see the rotation of an unknown dynamic object. This implies that a mathematically more complex human motion was accounted for before the remaining motion components could be used to infer an overall rotation. Our results indicate that the perceptual inference does not proceed in a hierarchical manner with the simpler components being identified first. Instead, prior knowledge acts as a starting point for the decomposition of an even relatively simple combination of two motions.

## Introduction

We rely on our visual system to produce a single and useful, if not necessarily veridical, representation of an outside world. However, the limited samples that we obtain via our sensory organs provide us with information which is both intrinsically incomplete and ambiguous. Our visual system appears to solve this problem by accumulating and exploiting a large store of expectations (prior knowledge) that describe statistical rules of the outside world^1–4^. However, the large number of potentially applicable Gestalts means that they themselves must be preselected based on the cues present in sensory evidence. Here, we examined the interplay between the two streams to explore the limits of the initial feedforward sweep and the kind of information it can extract without the assistance of the top-down system.

To this end, we used a point-light walker display, which is one of the best illustrations of how both bottom-up and top-down cues are used to reconstruct the visual scene^5, 6^; see Supplementary Video 2. In the point-light animation displays^7^, a handful of points is sufficient to create a vivid perception of a walking person, rich enough to identify a person or their gender from the movement^8, 9^. Perception of human motion is remarkably robust and is preserved even in noisy^10^, scrambled^11^, or rotating^12^ displays. One hallmark property of point-light walker perception, which highlights its reliance on the prior knowledge, is that it is largely abolished when the walker is presented upside-down^13^; please see Supplementary Video 3.

Here, we used this inversion effect to explore the limits of the feedforward computation. We employed four structure-from-motion displays that rotated around the vertical axis at 90°/s: Static and dynamic objects with no specifically recognizable shape plus an upright and an inverted point-light walker; see Supplementary Videos. Apart from the static object that served as a control, the motion of the individual dots was the sum of the common rotation and the dot-specific periodic motion relative to the rest of an object. Accordingly, we wondered whether the visual system would be able to decompose the resultant motion into the combination of an object-specific and rotation components; in other words, whether observers would perceive the overall rotation. In addition, we employed the inversion effect of the point-light walker displays to investigate how the foreknowledge about the object facilitates the global motion decomposition. To this end, we split participants into the *Naïve* and *Informed* groups. Both groups were instructed to report only on their perception of the global rotation. However, the latter were informed both about the presence of the human motion and about its orientation (upright or inverted), whereas the former were instructed that they would see various static and dynamic objects, with no further details provided. We report that for the dynamic objects, reliable perception of rotation depended both on the presence of an identifiable Gestalt (a point-light walker vs. a random shape) and on prior knowledge about its presence (*Naïve* vs. *Informed* groups).

## Results

### Main experiment

**Figure 1:**
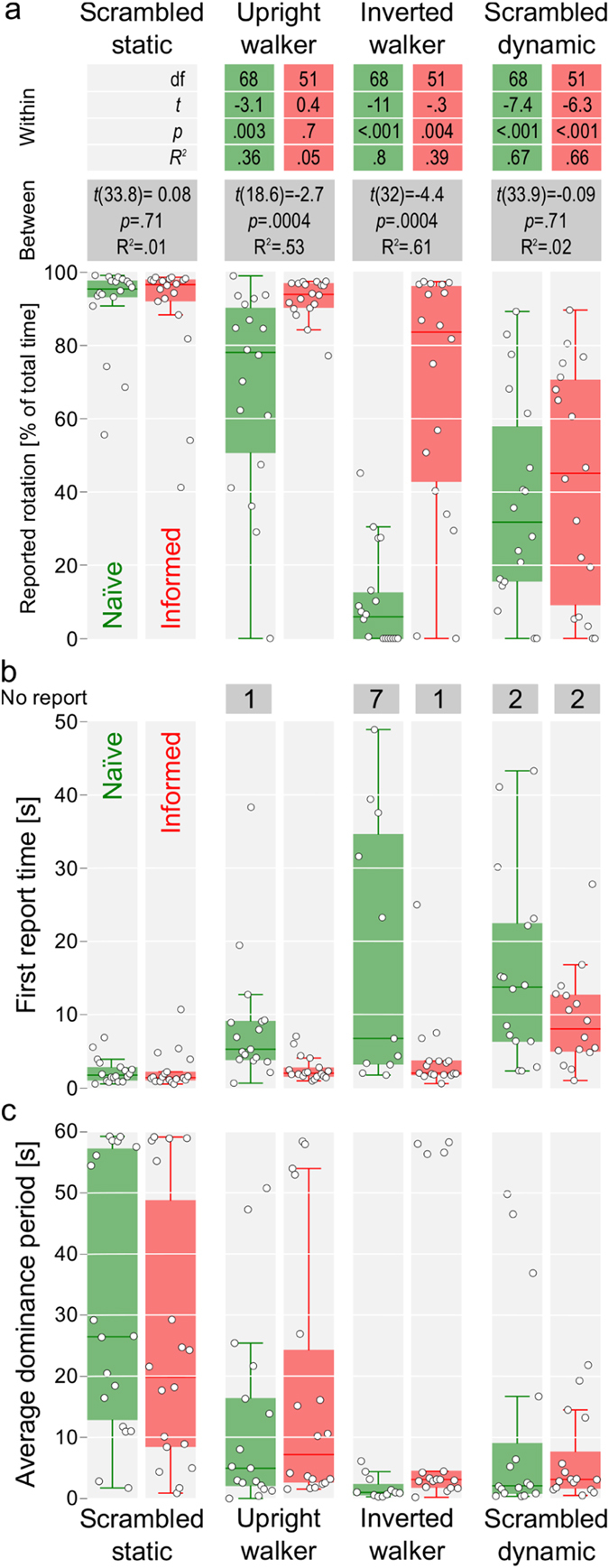
Results of the main experiment for the *Naïve* (green, left) and *Informed* (red, right) groups. Circles represent the individual observers. (**a**) Percentage of the total time when observers reported the perception of rotation. The *within* table above the plot shows the comparison of three conditions with dynamic objects to the *Scrambled static* condition. The comparison was performed separately for each group via a linear mixed model with the condition as a fixed effect and observers as a random effect. The *between* table above the plot shows the comparison for each condition *between the groups* using a paired permutation test. (**b**) Time of the first report. Numbers above the plot show the number of observers with no reports of the rotation. (**c**) Average dominance period duration (geometric mean).

In our main experiment, we examined how inversion of the point-light walker, as well as knowledge about the Gestalt’s presence, affected the perception of the rotation. To this end, observers viewed four ambiguously rotating structure-from-motion (SFM) displays: a static SFM shape (*scrambled static*, Supplementary Video 1), two that contained human motion (*upright* and *inverted walker* displays, see Supplementary Videos 2–3), and a scrambled human motion (*scrambled dynamic*, Supplementary Video 4). Observers were asked to report on whether they perceived the *rotation* and, if that was the case, to indicate its direction. The lack of reports indicated the absence of perceived rotation. Observers were split into *Naïve* and *Informed* groups and it was explained to both groups that the purpose of the study was to investigate whether motion, which was added to the individual flow elements, interferes with the perception of rotation. However, the observers from the *Informed* group were also informed about the nature of the displays that contained human motion. In addition, for this group the presentation of the *upright walker* preceded that of the *inverted walker*, giving them an opportunity to familiarize themselves with the display and the walker Gestalt. In contrast, the observers from the *Naïve* group were not informed about the presence of biological motion. The results of the experiment are presented in Fig. 1.

### Scrambled static

Observers from both groups had no trouble perceiving the rotation for the static SFM shape, which served as a control condition (*Scrambled static* in Fig. 1): all observers reported their first rotation percept within 11 seconds of the onset; 15 out of 18 observers in the *Naïve* group and 14 out of 18 observers in the *Informed* grouped reported seeing the rotation for more than 90% of the total viewing time.

### Upright walker

In contrast, the perception of rotation for the *upright walker* display clearly depended on the observers’ instructions. For the observers from the *Informed* group, the reports were not statistically different from the *scrambled static* condition (see Fig. 1a). They reported the onset of rotation within the first 7 seconds (Fig. 1b) and perceived it for most of the trial (all but one participant reported seeing the motion for more than 80% of trial). However, the observers from the *Naïve* group perceived the rotation for significantly less time compared to both the *Informed* group and to themselves in the *scrambled static* condition (Fig. 1a). This indicates that on the one hand, the non-linear perturbations, which the constant rotation added to the 2D projection of the point-light walker, were strong enough to interfere with the bottom-up mechanisms of the biological motion detection. On the other hand, the robust perception of the *Informed* group shows that the expectation of a biological motion and the associated top-down influence were sufficient to compensate for this disturbance.

### Inverted walker

The difference between the two groups was most pronounced for the *inverted walker* condition. Seven observers in the *Naïve* group failed to report any perception of rotation, as compared to just one in the *Informed* group. Moreover, ten observers in the *Informed* group reported perception for more than 80% of the time (more than expected^14^), as compared to the *Naïve* group, in which only one observer reported it for more than 40% of the time. Again, observers from the *Naïve* group were significantly different compared to either the observers from the *Informed* group or to themselves in the *scrambled static* condition (Fig. 1a). Furthermore, even for the *Informed* group, perception of the rotation was less stable than in the *scrambled static* condition, a stark contrast to the *upright walker* (Fig. 1). Taken together, these results indicate that the top-down mechanisms are critical for the perception of the *inverted walker*, enabling the visual system to disentangle the two motion components and observers to perceive the rotation as well.

### Scrambled dynamic

Finally, the *scrambled dynamic* condition was designed as a second control condition to provide no discernable Gestalt. Therefore, we were expecting to observe minimal reports of rotation. Nevertheless, knowledge about the potential existence of a Gestalt meant that a sizeable fraction of observers could coerce their visual system into grouping all dots into a single dynamic object, enabling them to perceive the rotation as well. However, this ability did not differ between the two groups (Fig. 1a) and observers’ average dominance period durations (Fig. 1c) show that they could maintain this perception only for brief periods of time, possibly reflecting the limits of the top-down control over perception.

### Learning experiment

**Figure 2:**
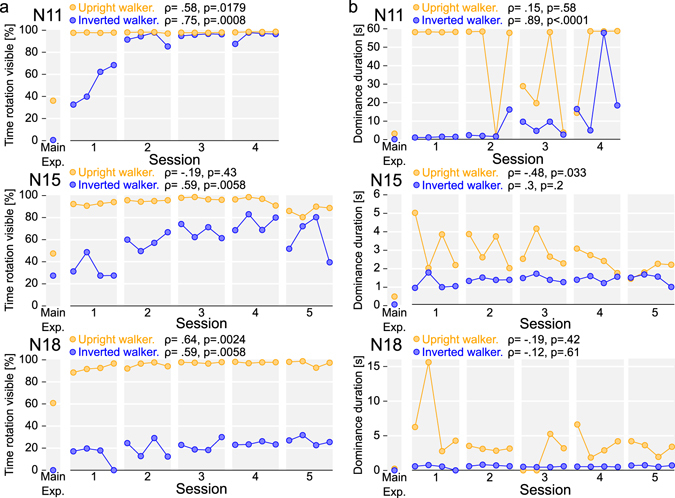
Results of Learning experiment. Values in the legend are Spearman’s rank correlation between a plot’s variable and time (running block index across all sessions). (**a**) The fraction of the time when observers reported the perception of rotation. (**b**) Average perceptual dominance duration (geometrical mean).

Since it has been shown that practice improves observers’ ability to detect the inverted human motion^15^, we recruited three observers from the *Naïve* group who had poor or no perception of rotation for the *inverted walker* condition, as well as having only a moderately reliable perception of rotation in the *upright walker* condition. The two observers (N15 and N18) returned for five additional sessions, the observer N11 for four sessions. These experimental sessions were performed on successive days and contained only *inverted* and *upright walker* conditions (four blocks per condition per session). Although all three already showed a reliable perception in the *upright walker* condition after the first session (Fig. 2a), only observer N11 quickly reached the same levels of rotation perception for the *inverted walker*. For observer N15 all five sessions were required, whereas observer N18, while showing a robust learning effect, could see the rotation for only ~20% of the total time even in the final session. Interestingly, an increase in the fraction of the time that observers reported the perception of rotation was not necessarily accompanied by an increased stability in the rotation dominance state (Fig. 2b). Moreover, although the duration of the dominance phases for the *inverted walker* became progressively more similar to those in the upright condition, the perception was still consistently less stable than in the *upright walker* condition (Fig. 2b). This indicates that although training made it easier for the observer to perceive the inverted walker Gestalt, its perception was still frequently interrupted, perhaps because of a temporary loss of concentration^16, 17^.

## Discussion

The key finding of the two experiments presented above is that in the absence of an identifiable Gestalt observers were also unable to perceive an overall rotation of the dynamic object. They did perceive it if a Gestalt could be identified via alternative cues, such as a form^18^ in the *upright walker* condition, if they had strong expectations about the nature of the dynamic object (*inverted walker* condition for the *Informed* group), or if they expected some kind of Gestalt to be present at all (*scrambled dynamic* condition).

In order for observers to perceive the rotation in the dynamic displays used here, the visual system needed to express the mathematically complex flow field motion as a combination of the overall rotation and the dot-specific periodic motion. The decomposition strategy is conceptually straightforward: If a component motion (e.g., the biological motion of the walker) is identified, it can be subtracted from the overall motion and the analysis can be repeated until no further decomposition is possible (or evident). This approach is used not only for motion but, for example, for color or auditory perception (there it is known as the “cancellation filter” approach^19^). However, as there are infinite combinations of various numbers of components adding up to the observed sensory inputs, the default option is to use an Occam’s razor. Thus, the decomposition is possible only if prior knowledge permits to presume the existence of more than one component. Here, it was the ability to recognize the biological motion Gestalt. For color perception, it is the deviation from the “gray world” assumption that allows the specific spectrum of the illumination source to be identified and subtracted^20^. In the auditory scene analysis, common onsets^21^ and harmonicity^22^ serve this purpose.

Current experimental results and the examples presented above reinforce the idea that sensory processing in general, and motion processing in particular, do not proceed in a hierarchical manner with the simpler components being identified first^23–25^. An overall rotation around the vertical axis (a constant angular motion in the 3D space) is mathematically simpler than biological motion, which consists of multiple time- and location-dependent combinations of a periodic rotational and translational motion. In the primate brain, rotation is associated with the hMT+/V5 region^26, 27^, whereas the perception of biological motion is correlated with activity in the posterior portions of the superior temporal sulcus^26, 28–30^, an area that could be considered to be of a higher order than hMT+/V5^31^. Accordingly, in purely hierarchical processing an overall rotation should be computed before the biological motion and, therefore, its representation and corresponding perception should not depend on the presence of the walker Gestalt. Instead, our results show that rotation appears to be perceived only if it is required to explain the remaining motion components once the more mathematically complex biological motion has been accounted for.

However, just as the feedforward approach is severely limited without access to prior knowledge, the top-down system relies on sensory evidence. Without it, the sheer number of potentially applicable Gestalts is too large to make a safe prediction. This turns perceptual inference into a chicken-and-egg problem: Cues in sensory evidence are needed to identify the relevant Gestalt, but knowledge about the Gestalt facilitates localization of those cues. Accordingly, the brain is likely to weight various kinds of sensory evidence^32^, perhaps by using prior knowledge to selects the specific processing mechanism or by starting the perceptual inference from islands of reliable representations^33^ and producing a reverberation between feed-forward and top-down streams^23^.

The way in which prior knowledge allowed observers to perceive the inverted rotating walker figure is also reminiscent of what has been demonstrated in other visual displays, which one may call “ambiguous”, such as puzzle pictures^34^, Mooney faces^35, 36^, and ambiguous (bi-stable) figures^37^. In these cases, a lack of prior knowledge about the existence of an alternative perceptual interpretation or about its nature also mostly precludes observers from experiencing it^38, 39^. Similarly to the perceptual learning of the walker gestalt, additional perceptual experience appears to facilitate switches in multi-stable displays^40, 41^.

## Conclusions

We conclude that perceptual inference does not proceed in a hierarchical manner with the simpler components being identified first. Instead, prior knowledge acts as a starting point for the decomposition of an even relatively simple combination of two motions.

## Methods

### Observers

All procedures were in accordance with the national ethical standards on human experimentation and with the Declaration of Helsinki of 1975, as revised in 2008, and were approved by the Otto-Friedrich-Universität Bamberg. All observers had normal or corrected-to-normal vision. Observers were naïve as to the purpose of the experiments. Informed consent was obtained from all subjects prior to the experimental session. The only condition for participation was the lack of prior experience with structure-from-motion displays. The participants were randomly assigned in two experimental conditions, so that the first 18 observers were assigned to the “Naïve” group, the other 18 to the “Informed” group.

### Apparatus

Displays were presented on a 24.1″ EIZO CG245W screen, with a refresh rate of 60 Hz and a spatial resolution of 1920 × 1200, with one pixel subtending approximately 0.029° at a viewing distance of approximately 50 cm.

### Displays

Observers were viewing ambiguously rotating bi-stable structure-from-motion (SFM) displays. SFM shapes rotated with an angular speed of 90°/s. Displays subtended approximately 10° vertically and 7° horizontally, individual dots subtending 0.15°. Dots were semi-transparent to exclude bias from the occlusions cues.

Four SFM shapes (one static, three dynamic) were used in the study and all were derived from a single dynamic walker sequence obtained from Vanrie & Verfaillie (2004)^42^. The same displays were used for all observers and conditions in both experiments. The walker, resampled for the 60 Hz presentation, was presented during the *Upright walker* (Supplementary Video 2) and *Inverted walker* (Supplementary Video 3) conditions. For the *Scrambled dynamic* condition (Supplementary Video 4), sequences of X, Y, and Z positions were shuffled between the dots, *i*.*e*. the motion trajectory of a dot would consist of the x-axis motion sequence from dot #5, the y-axis component from dot #2, and the z-axis motion sequence from dot #7 (components were shuffled without replacement, so that the same sequence was not repeated). This preserved the periodicity of dot motion but shuffled their positions and their motion relative to the other dots, scrambling the walker Gestalt. Finally, for the *Scrambled Static* condition, dot location was based on the first frame of the *Scrambled dynamic* movie (Supplementary Video 1).

### Procedure

For the main experiment, observers were divided into the *Naïve* (first 18 observers) and *Informed* (next 18 observers) groups. Both groups were told that the purpose of the experiment was to investigate whether alterations in the motion of individual flow elements could disrupt perception of the bi-stable rotation in SFM displays. Observers were instructed to continuously press either the left or right cursor keys if they perceived the *entire object* rotating with the front side going, respectively, to the left or to the right. They were told to abstain from pressing a key to indicate the lack of perceived rotation. The *Naïve* group was not informed about the nature of shapes used in the experiment. The *Informed* group was presented with the information about each shape and they were informed about the presence of the biological motion and its orientation before the corresponding conditions.

For the main experiment, a single session consisted of five blocks, each block lasting one minute. The condition order for the *Naïve* group was (1) *scrambled static* (training block, observers were encouraged to use that time to familiarize themselves with the display and the response procedure, data was not included in the analysis), (2) *scrambled static*, (3) *inverted walker*, (4) *upright walker*, (5) *scrambled walker*. For the *Informed* group, the *upright walker* display was presented before the *inverted walker*.

Three observers from the Naïve group participated in the *Learning* experiment. Two observers participated in five additional sessions and one observer in four additional sessions, which were performed on consecutive days. Each session consisted of four blocks of the *upright walker* (U) and four blocks of *inverted walker* (I) conditions. The presentation order was IUUIIUUI. Otherwise, the procedure was identical to that of the main experiment.

### Statistical analysis

Linear mixed models (package lmerTest^43^) with the condition as a fixed effect and observers as a random effect were used for within-group comparisons. Degrees of freedom were approximated using the Satterthwaite method. A custom code permutation independent sample t-test was used for between-groups comparison (10000 iterations, please refer to the analysis code in the repository), p-values were corrected for multiple comparisons using the Sidak adjustment.

### Data availability

All data files and the analysis code, which was used to produce figures and statistical comparison for the paper, are available under the CC-By Attribution 4.0 International license at https://osf.io/ynsvy/.

## Electronic supplementary material

Scrambled static display, please ensure that video is looped.

Upright walker display, please ensure that video is looped.

Inverted walker display, please ensure that video is looped.

Scrambled dynamic display, please ensure that video is looped.

Video legends
