# Supplementary material for: First, you need a Gestalt: An interaction of bottom-up and top-down streams during the perception of the ambiguously rotating human walker
Source: Sci Rep. 2017 Apr 25;7:1158. doi: 10.1038/s41598-017-01376-1 (PMC5430860; doi:10.1038/s41598-017-01376-1)
Supplement: Supplementary file 5 — Video legends [file 41598_2017_1376_MOESM5_ESM.doc]

# **First, you need a Gestalt: An interaction of bottom-up and top-down streams during the perception of the ambiguously rotating human walker**

**Alexander Pastukhov**^1,*^

^1^ Otto-Friedrich-Universität, Department of General Psychology and Methodology, Bamberg, 96047, Germany

^*^ pastukhov.alexander@gmail.com

# Video legends

Supplementary Video 1. Scrambled static display, please ensure that video is looped.

Supplementary Video 2. Upright walker display, please ensure that video is looped.

Supplementary Video 3. Inverted walker display, please ensure that video is looped.

Supplementary Video 4. Scrambled dynamic display, please ensure that video is looped.
